# Magnitude and determinants of multimorbidity and health care utilization among patients attending public versus private primary care: a cross-sectional study from Odisha, India

**DOI:** 10.1186/s12939-020-01170-y

**Published:** 2020-04-29

**Authors:** Sanghamitra Pati, Subhashisa Swain, J. André Knottnerus, Job F. M. Metsemakers, Marjan van den Akker

**Affiliations:** 1 Indian Council of Medical Research, Department of Health Research, ICMR Regional Medical Research Centre, Bhubaneswar, Chandrasekharpur, Bhubaneswar, Odisha 751023 India; 2 School of Medicine, Clinical Sciences Building, City Hospital, University of Nottingham, Nottingham, NG5 1PB UK; 3 Department of Family Medicine, School Caphri, Maastricht University, Maastricht, The Netherlands; 4 Academic Centre of General Practice / Department of Public Health and Primary Care, KU Leuven, Leuven, Belgium

**Keywords:** Multimorbidity, Health care utilization, Public primary care, Private primary care, Odisha, India

## Abstract

**Background:**

Multimorbidity in primary care is a challenge not only for developing countries but also for low and medium income countries (LMIC). Health services in LMIC countries are being provided by both public and private health care providers. However, a critical knowledge gap exists on understanding the true extent of multimorbidity in both types of primary care settings.

**Methods:**

We undertook a study to identify multimorbidity prevalence and healthcare utilization among both public and private primary care attendees in Odisha state of India. A total of 1649 patients attending 40 primary care facilities were interviewed using a structured multimorbidity assessment questionnaire collecting information on 22 chronic diseases, medication use, number of hospitalization and number of outpatient visits.

**Result:**

The overall prevalence of multimorbidity was 28.3% and nearly one third of patients of public facilities and one fourth from private facilities had multimorbidity. Leading diseases among patients visiting public facilities included acid peptic diseases, arthritis and chronic back pain. No significant difference in reporting of hypertension and diabetes across the facilities was seen. Besides age, predictors of multimorbidity among patients attending public facilities were, females [AOR: 1.6; 95% CI 1.1–1.3] and non-aboriginal groups [AOR: 1.6; 95%CI 1.1–2.3] whereas, in private females [AOR: 1.6; 95%CI 1.1–2.4], better socioeconomic conditions [AOR 1.4; 95% CI 1.0–2.1] and higher educational status [primary school completed [AOR 2.6; 95%CI 1.6–4.2] and secondary schooling and above [AOR 2.0; 95%CI 1.1–3.6] with reference to no education were seen to be the determinants of multimorbidity. Increased number of hospital visits to public facilities were higher among lower educational status patients [IRR: 1.57; 95% CI 1.13–2.18] whereas, among private patients, the mean number of hospital visits was 1.70 times more in higher educational status [IRR: 1.70; 95%CI 1.01–3.69]. The mean number of medicines taken per day was higher among patients attending private hospitals.

**Conclusion:**

Our findings suggest that, multimorbidity is being more reported in public primary care facilities. The pattern and health care utilization in both types of settings are different. A comprehensive care approach must be designed for private care providers.

## Background

One of the greatest challenges that health systems will face globally in the twenty-first century is the increasing burden of chronic diseases [1]. Driven by increasing longevity, the presence of multiple (more than one) chronic conditions, commonly referred to as ‘multimorbidity’ is progressively more frequent among individuals [2, 3]. Presence of multimorbidity leads to frequent health care consultations, longer hospital stays, poorer health-related quality of life (HRQOL), increased health care costs and higher mortality [4–7]. Multimorbidity is increasingly being identified as one of the most pressing challenges for the health care system owing to its adverse health and economic implications and for health care workers, whose decision making is generally supported by single disease-specific guidelines [8, 9].

Several studies in high income countries have demonstrated the magnitude of multimorbidity to be emerging; with the prevalence varying from 25 to 60% in health care and community settings [10–13]. In contrast, the situation of multimorbidity in low and middle income countries (LMICs) is unclear [14–19]. Amongst LMICs, India, the second largest demography in the world, is witnessing an upward shift in life expectancy, with non-communicable diseases (NCDs) replacing infectious illnesses as the dominant contributors to morbidity and mortality [20, 21]. Despite the growing burden of chronic conditions, there is very limited knowledge on the occurrence multimorbidity till date principally owing to lack of basic epidemiologic data. Our recent systematic review on multimorbidity indicated the research on this topic to be in its infancy in India with most of the studies restricted to the elderly population and no reports available from primary care settings [18]. This is a critical knowledge gap, since primary care is the first and most frequently consulted health care facility and constitutes the scaffold of health care delivery. Primary care practice by virtue of its continuity, comprehensiveness and coordination is the most ideal setting for delivering optimal care needed by patients with multiple chronic conditions [22, 23]. Evidence demonstrates that prevention and control of chronic diseases by primary care interventions focusing on those at high risk and those with established diseases are more cost-effective when compared to secondary and tertiary care interventions [24].

Currently, in India, primary care is being delivered by both public and private systems [25]. The public healthcare sector is heavily funded by the government and patients pay a nominal sum toward buying drugs, diagnosis or for the treatment [26]. While the private healthcare service is fee for service where patients pay out-of-pocket themselves, or it is funded by their employers or by insurance companies. Even though the healthcare services provided by private sectors in India is ill studied, the report by Basu et al., has provided some insight into it [27]. Despite the higher cost of private health care, the recent national sample survey indicates that 70% of the patients in India avail of private health care in addition to or in parallel to public health services [28]. Mostly, the primary health care system is oriented towards the care of acute, episodic illnesses as well as maternal and child health. In view of the emerging burden of NCDs, the ministry of Health and Family Welfare (MOHFW) has recently initiated national control program for NCDs with defined role of primary care [26]. However, chronic disease management is still largely being done in a sporadic, unplanned and uncoordinated manner by the primary care practitioners and the control strategies and clinical guidelines are focused on the management of single conditions. Therefore, an epidemiological understanding of the multimorbidity situation in both public as well as private primary care settings, and the differences between these, is necessary to help guide effective realigning of the non-communicable disease program and designing appropriate primary care protocols [29].

We undertook the first ever study in India to determine and compare the multimorbidity prevalence in patients attending public and private primary care clinics and their health care utilization and their determinants in these two settings. Our research question was “Is there a difference in the profile (characteristics and determinants) of multimorbidity between patients attending public and private primary care?” In addition to depicting differences in prevalence and correlates of multimorbidity among patients attending public vis-à-vis private primary care facilities, we also considered health care utilization (in terms of physician consultation and number of drugs being consumed).

## Methods

### Study design and participants

We undertook a cross-sectional study from October 2013 to March 2014 in 40 primary care facilities (20 public and 20 private) in Odisha state of India [19]. Odisha is a province located in the eastern coast of India and has health and socio-demographic indicators similar to the national average. A two-stage clustered stratified random sampling method was adopted for recruiting health facilities. As the community health centers (CHC) are the cornerstone of the Indian health care system through provision of both preventive and curative primary care to patients, we decided to conduct our study in public and private community health centers. In the first stage, 30 districts of the state were divided into two clusters, i.e. economically well-developed [20] and less-developed [10], as per the state specific guideline [30]. From each cluster, districts were selected using stratified sampling methods, comprising four districts from less-developed and six from the developed districts. From each district, two community health centers (CHC) were randomly selected. For every CHC, a corresponding private facility in the same vicinity/region providing similar services was randomly included thus totaling to 20 private primary care facilities [31]. The schematic presentation of sampling is provided in Additional file 1.

As we did not have studies from India on prevalence of multimorbidity, the sample size was calculated based on our pilot study while validating the multimorbidity assessment tool [32]. Considering that 23% of patients attending primary health care settings have multimorbidity [32], the sample size was estimated to be 1456 within relative precision error of 12.5% of prevalence considering the design effect of 1.7. After accounting for 13% non- response based on our pilot experience [32], the final intended sample size was 1670. For comparison, it was decided to divide this sample number equally between private and public health care facilities, by stratified sampling. The number of patients for each facility was calculated based on respective outpatient attendance. Patients were recruited by systematic random sampling from the list of patients attending the health facilities. Those selected patients were interviewed only after the consultation with the doctors to avoid any disturbance to the hospital patient management system and delays. We included patients aged 18 years or above attending the facilities, who provided the consent. Patients too ill to participate, those with insufficient cognitive ability to complete the questionnaire and those with debilitating physical disabilities and mental disabilities and not willing to participate were excluded from the study. Also, the exit interviews helped us to record more diagnosis and verify the self-reported diagnosis going through the prescriptions. To avoid duplication from both the facilities, unique identification numbers were given to the patients and who have already been interviewed in any of the facility previously under the present study were excluded. Interviews were conducted by four well trained field investigators with a nursing background well versed with local language and patient history taking. Each interview spanned from 20 to 30 min [32].

### Data collection

To collect data, we developed and validated a structured tool - Multimorbidity Assessment Questionnaire for Primary Care (MAQ-PC) which was translated into the vernacular language (Odia). With no gold standard available to measure multimorbidity in India, we followed an iterative process to design this comprehensive tool. The detailed methodology for development and validation of our tool is available elsewhere [32]. In short, the multimorbidity subscale explored the presence of any of the 18 listed self-reported chronic diseases. Open options for “any other conditions” were added to capture unlisted conditions if any. Three more chronic diseases (hypotension, eczema and psoriasis) were extracted from the additional list and added to the previous list of 18, thus totaling to 21 chronic diseases (Additional file 2). We followed the prescribed guidelines for analysis of PHQ-9 towards diagnosing depression, and a score of 10 or more was taken as a cutoff value for depression, considering non-reporting of psychiatric patients in primary care in Indian context [33]. Additionally, we elicited health care use in terms of number of outpatient visits and inpatient admissions in the last 12 months per person per year and count of medications per person per day.

### Ethical approval

The study adhered to the Declaration of Helsinki principles and was approved by the Institutional Ethics Committee of Public Health Foundation of India, New Delhi (Vide no. TRC-IEC-173/13). Respective physicians in charge of the health facilities were contacted and their permission was obtained in prior. Written informed consent was obtained from all respondents following an explanation of the study aims and procedures. Necessary steps were taken to preserve patient anonymity and confidentiality.

### Data analysis

Analysis was carried out using sampling weight, which was calculated taking account of the complex nature of the sample, i.e. different sampling fraction owing to difference in patient load/visited each CHC or private facility and clustering by facility using the ‘svy’ command in STATA (Version 12.0, Stata crop, TX). We defined ‘multimorbidity’ as the presence of two or more co-occurring chronic or long term diseases or conditions [2].

Separate analysis was done for public and private patient groups. Age was categorized into 6 groups (18–29, 30–39, 40–49, 50–59, 60–69 and > =70 years). We categorized the socioeconomic variable based upon the status as ‘above poverty line’ (APL) and ‘below poverty line’ (BPL). This rank has been introduced by the Government of India, and each family identified as BPL has been provided with a card, which was verified during the survey [30]. Descriptive analysis of socio-demographic factors; age, sex, socioeconomic status (APL/BPL), ethnicity (aboriginal, non-aboriginal), marital status (single or married), education (no schooling, primary completed, secondary and above) was carried out. Aboriginal group includes tribal and population of schedule caste, as per the Government’s law. Additionally, we elicited health care use in terms of number of outpatient visits and inpatient admissions in the last 12 months per person per year and count of medications per person per day. Mann-Whitney test was performed to find the statistical difference across the facilities.

We calculated age- and sex adjusted prevalence of multimorbidity across socio-demographic variables and used binary logistic regression analysis to estimate adjusted odds ratios (OR) of the association of various socio-demographic determinants with multimorbidity. A linear trend test was performed to investigate whether the probability of having multiple chronic conditions varied across the age-groups and by sex. Mean and median number of medicines taken per day and hospital visits in last 1 year were calculated across the age groups for males and females in both groups. Among patients with multimorbidity, the associated factors for health care use were explored using zero inflated negative binomial regression analysis and expressed as incidence rate ratio (IRR), separately for public and private group.

## Results

**Fig. 1:**
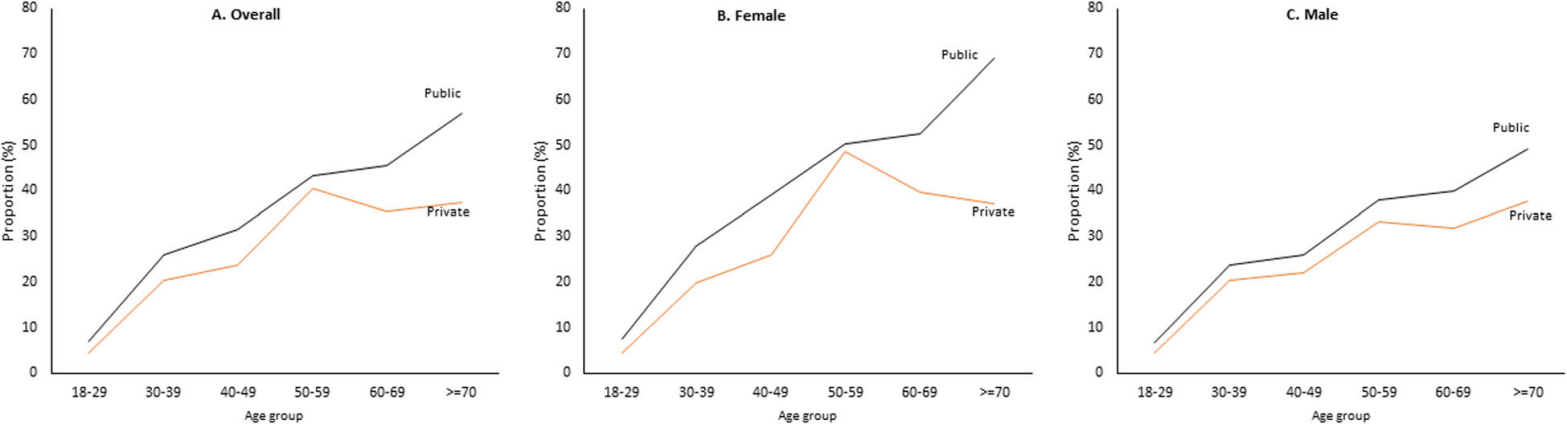
Prevalence of multimorbidity across age group. The prevalence of multimorbidity shows a trend of linear increase along with age in both the sexes for patients attending public health facilities. Among patients visiting private facilities, the prevalence trend declined after 60 years of age in females only. In both sexes and facilities, the mean numbers of morbidities were seen to increase linearly with age

In total 1649 patients from both public (*n* = 849) and private (*n* = 800) facilities agreed to participate (response rate 98.75%) in the study. In both groups the proportions of males, aboriginal group, and below poverty line were more compared to the proportions of females, non-aboriginals and above poverty line, respectively. The mean age of participants was 44.5 years (standard deviation: 15.92) and 44.83 years (standard deviation 16.29) in public and private facilities, respectively. The overall prevalence of multimorbidity was 28.3% [95%CI 25.9–30.7%]. Nearly one third of patients from public facilities [30.7%; 95%CI 27.4–33.9%] and one fourth from private facilities [24.6%; 95%CI 21.3–28.0%] had multimorbidity. Details of the socio-demographic distributions and proportion of multimorbidity are presented in (Table 1). Figure 1 shows age and sex wise distribution of multimorbidity in public and private faciltities.

**Table 1:** Socio-demographic distribution and percentage of mulitmorbidity

Characteristics	Public (*n* = 849)		Private(*n* = 800)		Total (*n* = 1649)	
	Weighted(%) [95%CI]	Multimorbidity % [95%CI]	Weighted% [95%CI]	Multimorbidity % [95%CI]	Weighted% [95%CI]	Multimorbidity% [95%CI]
Age Group^a^						
18–29	23.4 [20.4–26.3]	7.1 [3.5-10.6]	21.2 [18.2–24.3]	3.4 [0.7–6.13]	22.6 [20.4–24.7]	5.8 [2.0–9.6]
30–39	17.9 [15.2–20.6]	23.7 [16.9–30.5]	18.4 [15.4–21.4]	18.6 [12.2–24.9]	18.1 [16.1–20.1]	22.2 [15.1–29.4]
40–49	20.1 [17.3–22.9]	30.3 [23.4–37.3]	20.1 [17.9–24.0]	22.5 [16.3–28.6]	20.5 [18.4–22.5]	24.3 [17.7–30.9]
50–59	16.3 [13.7–18.8]	43.5 [35.2–51.8]	17.4 [14.5–20.4]	35.9 [27.6–44.3]	16.7 [14.8–18.7]	36.2 [27.9–44.5]
60–69	14.8 [12.4–17.3]	47.2 [38.5–55.9]	14.0 [11.4–16.7]	34.8 [25.9–43.9]	14.6 [12.7–16.4]	36.9 [28.1–45.8]
> =70	7.4 [5.6–9.2]	54.5 [42.4–66.6]	7.7 [5.8–9.7]	35.4 [23.6–47.1]	7.5 [6.2–8.9]	44.4 [33.0–55.8]
Gender^a^						
Male	54.6 [51.1–58.0]	27.0 [25.7–28.4]	57.6 [53.8–61.3]	26.5 [25.2–27.8]	55.8 [53.2–58.3]	25.1 [22.1–28.0]
Female	45.4 [41.9–48.9]	25.5 [23.9–26.7]	42.4 [38.6–46.1]	26.9 [25.3–28.4]	44.2 [41.6–46.8]	32.5 [29.0–35.9]
Ethnicity						
Aboriginal	30.3 [27.1–33.5]	26.5 [26.2–26.9]	24.5 [21.3–27.6]	26.5 [26.0–26.9]	28.0 [25.7–30.3]	27.7 [26.3–29.2]
Nonaboriginal	69.7 [66.5–72.9]	26.6 [26.3–26.8]	75.5 [72.4–78.6]	26.3 [26.1–26.6]	71.4 [69.7–74.3]	28.5 [27.6–29.4]
Socio Economic Status						
Below Poverty Line	61.8 [58.4–65.2]	26.4 [26.1–26.7]	61.2 [57.5–65.0]	26.6 [26.3–26.9]	61.6 [59.1–64.1]	28.8 [27.8–29.7]
Above Poverty Line	38.2 [34.7–41.6]	26.3 [25.9–26.7]	38.7 [35.0–42.5]	26.5 [26.2–26.9]	38.4 [35.8–40.9]	27.5 [26.2–28.8]
Highest Education						
No Schooling	36.9 [33.5–40.3]	27.5 [27.1–27.8]	40.7 [36.9–44.4]	27.1 [26.7–27.5]	38.1 [35.8–40.9]	35.0 [33.7–36.3]
Primary	32.4 [29.1–35.6]	26.2 [25.8–26.6]	28.2 [24.8–31.5]	26.2 [25.7–26.6]	30.7 [28.3–33.1]	28.3 [27.1–29.5]
Secondary and above	30.7 [27.5–33.9]	25.8 [25.5–26.2]	31.1 [27.5–34.7]	25.6 [25.2–26.0]	31.1 [28.5–33.3]	20.1 [19.6–21.1]
Marital Status						
Single	20.5 [17.7–23.4]	27.1 [26.6–27.6]	19.6 [16.6–22.6]	26.8 [26.3–27.3]	20.2 [18.1–22.2]	29.3 [28.5–30.1]
Married	79.4 [76.6–82.3]	26.4 [26.2–26.7]	80.4 [77.4–83.4]	26.3 [26.0–26.5]	79.8 [77.7–82.0]	24.3 [22.0–26.6]
Total	849 [100]	30.7 [27.4–33.9]	800 [100]	24.6 [21.3–28.0]	1649 [100]	28.3 [25.9–30.7]

^a^The prevalence of multimorbidity across age-group was adjusted for sex, and across sex was adjusted for age; for others prevalence was adjusted for age and sex

The distribution of chronic conditions and number of morbidities across the facilities are given in Additional file 2. Top leading conditions reported were acid peptic disorder (31%), hypertension (16.4%) and arthritis (15.4%). Similar pattern was seen in both public and private facilities. Chi-square test reveals, compared to private facilities, patients visiting public facilities had a higher prevalence of acid peptic diseases, arthritis, chronic back pain and tuberculosis, which was statistically significant at *p*-value < 0.05. Whereas, number of people having hypertension, diabetes, chronic lung diseases and other chronic conditions visiting health facilities were indifferent (Additional file 2). The proportion of patients with single morbidity was higher in private facilities [28.5%], whereas the proportion of patients having two morbidities [18.1%] and more than two morbidities [12.6%] were higher in public facilities, which was statistically significant (Additional file 2). The prevalence of multimorbidity shows a trend of linear increase along with age in both the sexes for patients attending public health facilities. Among patients visiting private facilities, the prevalence trend declined after 60 years of age in females only.

The determinants of multimorbidity were estimated separately for public and private patients using binary logistic regression methods. In both facilities, age was found to be the strongest predictor of multimorbidity. Besides age, among patients attending public facilities, females [AOR: 1.6; 95% CI 1.1–2.3] and non-aboriginal groups [AOR: 1.6; 95%CI 1.1–2.3] had higher odds of having multimorbidity compared to males and aboriginal counterparts, respectively. Whereas, in private facilities, females [AOR: 1.6; 95%CI 1.1–2.4], better socioeconomic conditions [AOR 1.4; 95% CI 1.01–2.1] and higher educational status [primary school completed (AOR 2.6; 95%CI 1.6–4.2) and secondary schooling and above (AOR 2.0; 95%CI 1.1–3.6)] with reference to no education were seen to be the determinants of multimorbidity after adjusting for other variables (Table 2).

**Table 2:** Factors associated with multimorbidity in private and public facilities

Characteristics	Public (*n* = 849)		Private(*n* = 800)	
	Unadjusted^a^ OR[95%CI]	Adjusted OR^b^ [95%CI]	Unadjusted OR^a^ [95%CI]	Adjusted OR^b^ [95%CI]
Age Group (in years)				
18–29	Reference	Reference	Reference	Reference
30–39	4.62 [2.34–9.14]*	6.70 [2.89–15.57]*	5.47 [1.99–15.00]*	6.13 [2.06–18.21]*
40–49	6.11 [3.16–11.80]*	9.37 [4.05–21.64]*	6.70 [2.54–17.66]*	8.73 [2.93–25.96]*
50–59	10.07 [5.20–19.48]*	16.73 [7.12–39.31]*	14.66 [5.54–38.78]*	19.42 [6.52–57.80]*
60–69	11.06 [5.67–21.56]*	17.21 [7.35–40.28]*	11.90 [4.43–31.92]*	16.48 [5.45–49.83]*
> =70	17.64 [8.31–37.64]*	26.29 [10.52–65.66]*	12.93 [4.55–36.69]*	20.73 [6.54–65.67]*
Gender				
Male	Reference	Reference	Reference	Reference
Female	1.39 [1.03–1.89]*	1.59 [1.11–2.27]*	1.30 [0.90–1.88]	1.61 [1.07–2.42]*
Ethnicity				
Aboriginal	Reference	Reference	Reference	Reference
Nonaboriginal	2.02 [1.31–3.12]*	1.56 [1.06–2.32]*	1.60 [1.14–2.26]*	1.52 [0.95–2.45]
Socio Economic Status				
Below Poverty Line	Reference	Reference	Reference	Reference
Above Poverty Line	1.19 [0.87–1.63]	1.30 [0.90–1.86]	1.68 [1.16–2.42]*	1.35 [1.01–2.06]*
Highest Education				
No Schooling	Reference	Reference	Reference	Reference
Primary	0.96 [0.67–1.36]	1.26 [0.83–1.93]	1.83 [1.19–2.81]*	2.59 [1.59–4.23]*
Secondary and above	0.54 [0.36–0.79]	1.21 [0.72–2.05]	1.01 [0.64–1.60]	1.99 [1.11–3.55]*
Marital Status				
Single	Reference	Reference	Reference	Reference
Married	1.04 [0.71–1.52]	0.61 [0.36–1.01]	2.39 [1.38–4.16]*	0.93 [0.49–1.78]

* *P* value < 0.05

^a^Univariate logisitic regression

^b^Multivariable logistic regression

**Fig. 2:**
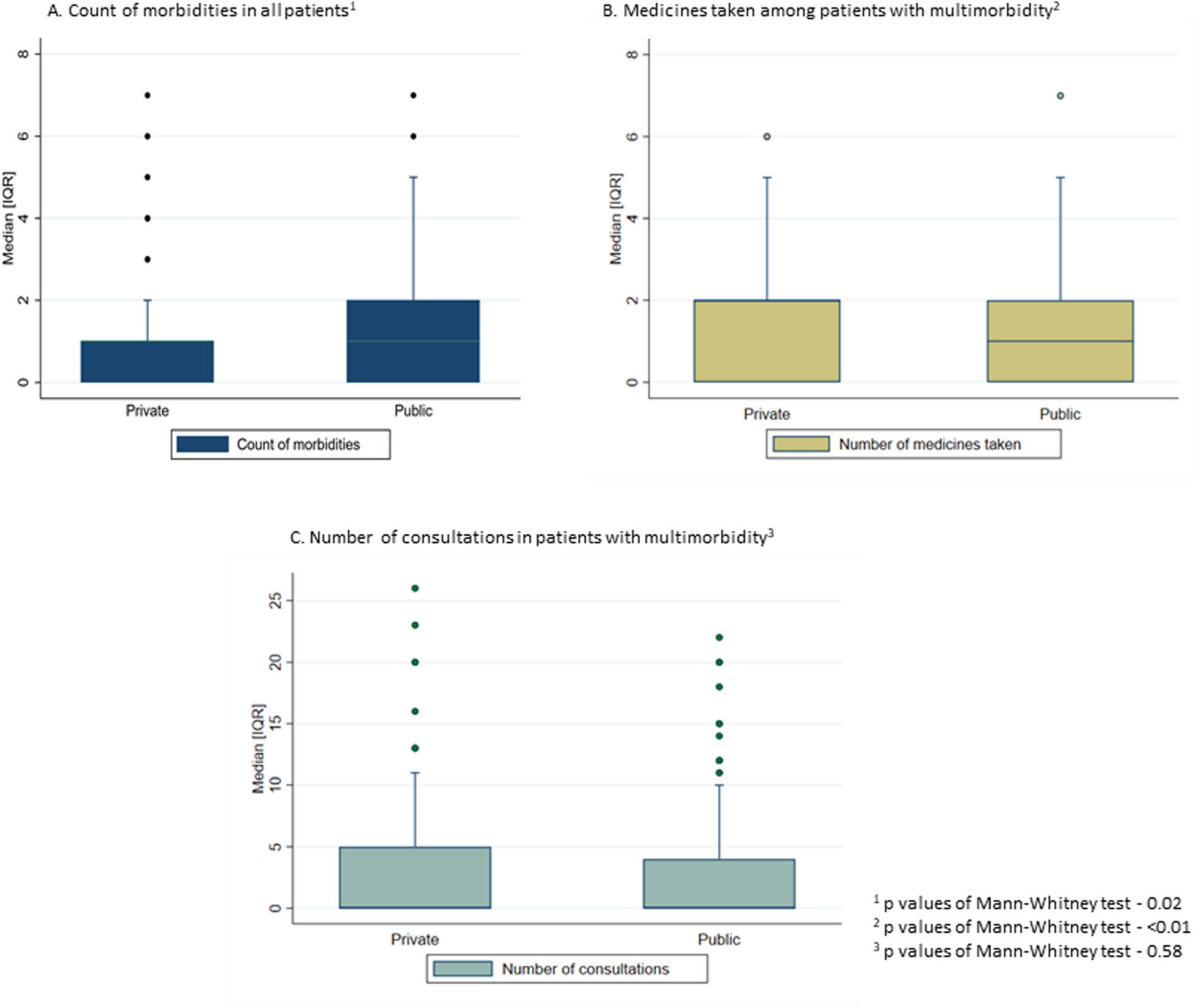
Mean number of morbidities, consultations medicine use among patients attending public and private facilities. The distribution of mean number of morbidities, medicines taken, and consultations were plotted across public and private facilities

The mean of number of chronic conditions was 1.01 and 0.87 in public and private facilities, respectively. Mean number of consultations among patients with multimorbidity was nearly 2.7 in both the facilities. The mean number of medicines used in multimorbidity patients from public facility was 1.2 and those visited private facility was 2.7. Details of these distribution is provided in Fig. 2a, b and c. There was statistical difference across the facilities for number of morbidities and medicine use at *p* value < 0.05.

**Fig. 3:**
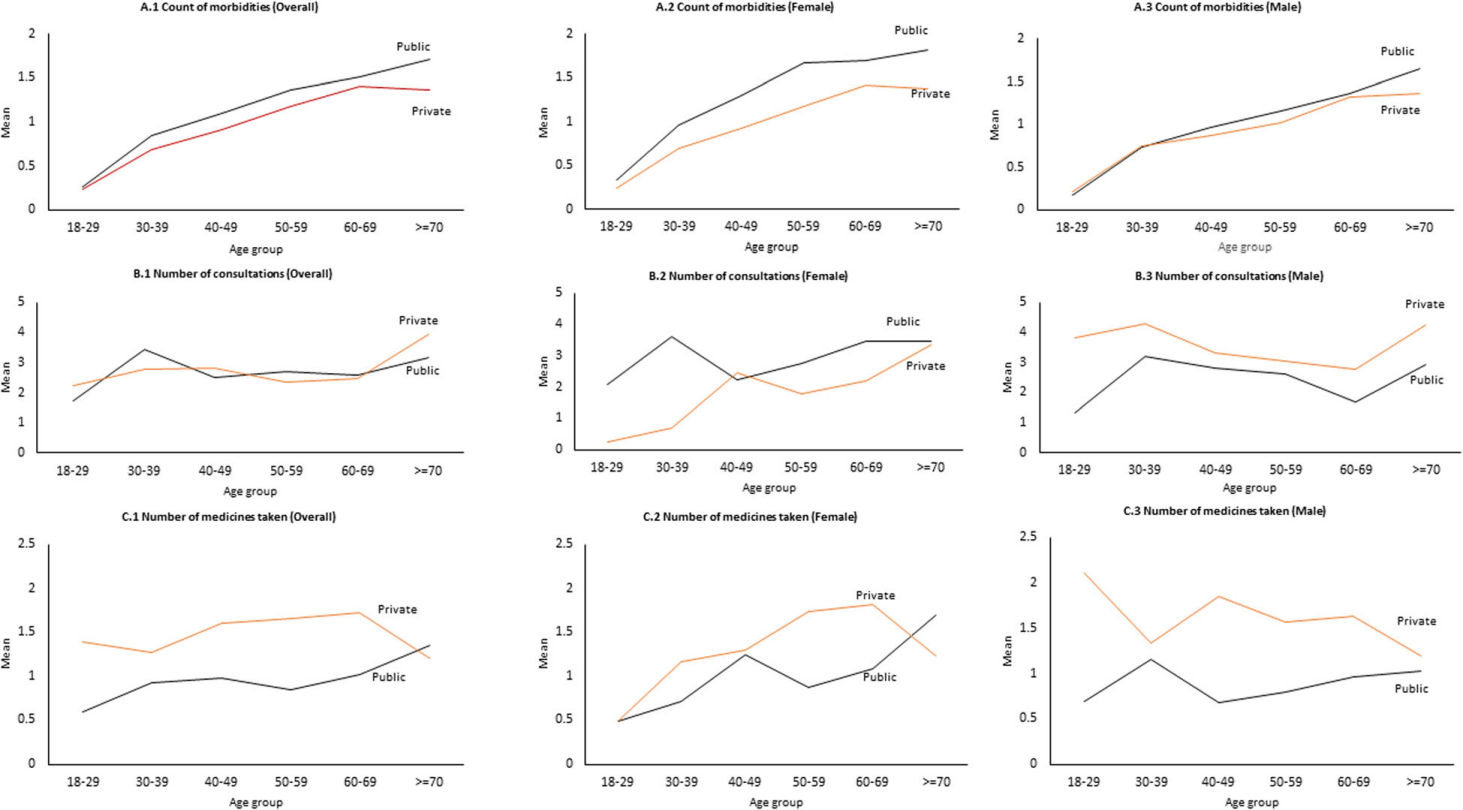
Mean number of morbidities, consultations medicine use among patients attending public and private facilities across the age group and gender. The distribution of mean number of morbidities, medicines taken, and consultations were plotted across the age group for both genders

The distribution of mean number of morbidities for all patients, medicines taken and consultations among multimorbidity patients were plotted across the age group for both genders. (Fig. 3) Overall distribution of mean hospital visits across facilities were similar. (Fig. 3B.1) However, the mean number of hospital visits per person per year among female multimorbidity patients attending public facilities was higher than private facilities. (Fig. 3B.2) Whereas, in males, hospital visits was higher in all age group in multimorbidity patients attending private facilities (Fig. 3B.3). Among multimorbidity patients attending public facilities, the number of hospital visits were 1.57 times higher among those with lower educational status [IRR: 1.57; 95% CI 1.13–2.18] and exhibited a significant association with age group, whereas, among private patients, the mean number of hospital visits was 1.70 times more in higher educational status [IRR: 1.70; 95%CI 1.01–3.69]. The mean number of medicines taken was found to be more in multimorbidity patients visited private facilities across all age group and gender (Fig. 3C.1, C.2 and C.3). In female multimorbidity patients attending public facilities, the medicine count increases after the age of 50–59 and in male counterparts, it increased after the age 40–49 years (Fig. 3C.2 and C.3). In multimorbidity patients from private facilities, the number of medicines taken decreases after the age of 60 in both the gender. Non-aboriginal patients attending public health facilities use medicines for chronic conditions 1.18 times more than aboriginal group of patients. Among patients attending private facilities, those belonging to non-aboriginal group and those with higher socioeconomic status consumed medicines 1.78 and 1.36 times more than aboriginal and lower socioeconomic group of patients, respectively (Table 3).

**Table 3:** Factors associated with health care utilization among multimorbid patients from public and private facilities

Characteristics	Public (*n* = 261)		Private(*n* = 200)	
	Number of hospital visits Adjusted IRR^a^ [95% CI]	Number of medicines taken Adjusted IRR^a^ [95% CI]	Number of hospital visits Adjusted IRR^a^ [95% CI]	Number of medicines taken Adjusted IRR^a^ [95% CI]
Age Group (in years)				
18–29	Reference	Reference	Reference	Reference
30–39	3.85 [2.05–7.24]^*^	1.23 [0.58–2.60]	1.42 [0.44–4.52]	1.04 [0.37–2.92]
40–49	3.56 [1.94–6.56]^*^	1.38 [0.63–3.02]	2.13 [0.60–7.67]	1.36 [0.52–3.53]
50–59	3.63 [2.16–6.09]*	1.28 [0.58–2.83]	1.69 [0.54–5.25]	1.36 [0.52–3.57]
60–69	3.84 [2.23–6.59]^*^	1.43 [0.67–3.07]	1.68 [0.44–6.46]	1.34 [0.49–3.62]
> =70	2.98 [1.62–5.49]^*^	1.60 [0.75–3.41]	1.96 [0.55–6.96]	1.10 [0.40–3.02]
Gender				
Male	Reference	Reference	Reference	Reference
Female	1.08 [0.72–1.61]	0.98 [0.70–1.37]	0.92 [0.56–1.51]	0.85 [0.62–1.15]
Ethnicity				
Aboriginal	Reference	Reference	Reference	Reference
Non-aboriginal	1.24 [0.84–1.83]	1.18 [1.02–1.69]^*^	0.81 [0.53–1.26]	1.78 [1.04–3.02]^*^
Socio Economic Status				
Below Poverty Line	Reference	Reference	Reference	Reference
Above Poverty Line	0.99 [0.73–1.34]	1.04 [0.80–1.36]	0.95 [0.56–1.61]	1.36 [1.01–1.86]^*^
Highest Education				
No Schooling	Reference	Reference	Reference	Reference
Primary	1.57 [1.13–2.18]^*^	0.74 [0.54–1.02]	1.15 [0.7–1.89]	0.99 [0.71–1.38]
Secondary and above	1.29 [0.80–2.09]	1.05 [0.72–1.52]	1.70 [1.01–3.69]^*^	0.92 [0.62–1.38]
Marital Status				
Single	Reference	Reference	Reference	Reference
Married	0.85 [0.59–1.21]	0.79 [0.52–1.21]	0.69 [0.30–1.57]	0.80 [0.50–1.28]

*CI* Confidence interval, *IRR* Incidence Rate Ratio

*P value < 0.05

^a^Multivariable Zero inflated negative binomial regression. Each variable was adjusted for other covariates in the table

## Discussion

The available studies on multimorbidity are mostly from developed countries, using databases from primary care [18]. However, very little research on this topic has been undertaken in LMICs, where 80% of the burden of NCDs falls [18]. Few recently published reports on multimorbidity in LMICs are either community based or restricted to a limited number of public facilities, which does not capture the true extent of multimorbidity and a clear picture from private health care facilities is lacking. India, a rapidly urbanizing country is currently entangled with high burden of NCDs [21]. As per the latest national survey, nearly half of the people in India avail private health care services in conjunction or parallel to public health care services for chronic diseases [28]. In our study, more than half of study patients (54.7%) had at least one chronic condition and around one-third had multimorbidity. The most common conditions reported were acid peptic disease, hypertension, arthritis, chronic back pain, vision impairment and diabetes. Nearly 30 and 25% patients visited public and private facilities had multimorbidity, respectively. Along with age and gender, in public facilities multimorbidity was 1.5 times more among non-aboriginal patients, whereas, in private facilities, it was associated with socioeconomic status of the patient. Hospital visits among multimorbidity patients in public and private facilities were associated with education and medicine intake was associated with ethnicity and socioeconomic status of the patients.

The sample characteristics are similar to the population structure of the state (Additional file 3). However, the observed minimum variance was due to the health seeking behavior of the people in the states. Previous small sample studies conducted in Bangladesh and India have identified a prevalence of multimorbidity of 53.8 and 77% in persons aged more than 60 years, respectively [15, 16]. Our study revealed that approximately one-fourth in private care had multimorbidity, and most patients had either no or single chronic condition. The lower prevalence of multimorbidity and a higher prevalence of mono-morbidity in private facilities could have been due to lower availability of specialists and supporting services [34, 35]. Many patients preferred private health services with the assumption of receiving better and timely care even though they are costlier than public medical facilities. However, when these illnesses become chronic long-term, patients chose to shift the treatment for continued care at public health facilities [34–36]. Further, a significant variation in prevalence of multimorbidity (sex adjusted) across different age groups was seen between public and private facilities. Nearly half of the elderly patients (aged 60 years or more) attending public facilities had multimorbidity while majority of middle-aged people (50–59 years) attending private facilities had multimorbidity. Availability of basic diagnostic services such as blood pressure measurement and blood sugar testing without any cost at public primary care centers and distribution of free medicines and consultations at public health facilities could be few of the factors to attract more patients with multimorbidity, particularly those from older age groups [25]. High medical costs for elderly people at private healthcare facilities could lead to personal financial problems. However, free diagnostics, treatment and supporting services motivates elderly to seek care at public health care facilities; which helps in bridging the gap in elderly inequity.

In alignment with many studies [18, 27], in addition to age, we observed higher risk of multimorbidity in women and non-aboriginal groups seeking care from public facilities. Given the lower health care seeking behavior among females, the reasons for such figure merits further exploration in India [21, 36]. As explained earlier, accessibility and affordability might be responsible for reporting multimorbidity more often in non-aboriginal people visiting public health facilities, since most of them are residing in remote-inaccessible areas, while people with higher socioeconomic status and educational status might prefer private over public facilities. Various studies investigating the distribution of multimorbidity have directed attention towards the possible interplay of social and economic deprivation [30, 37–40]. In high income countries, persons with low socioeconomic status are more likely to have multimorbidity as compared to their affluent counterparts [12]. In contrast to findings from western countries, we detected neutral association among public facilities and strong positive association between income and multimorbidity in private facilities. Such positive associations have been reported from other LMIC [15–18]. This may be related to the fact that patients of lower socio-economic status are less likely to seek health care and therefore less likely to have chronic diseases diagnosed. Also, with higher income the affordability is better, for which patients prefer to visit private facilities. Patients who were using private healthcare were able to spend more on travel, food and accommodation than those who were using public healthcare. The difference between health care seeking behaviors among different socioeconomic groups may contribute to the health care discrepancy [12–15]. It is evident that patients with multimorbidity visit health care facilities more and spend more on treatment [40]. In India, private service providers play a crucial role in providing health care services but with a wide variety of costs and quality. However, there is lack of standard guideline/regulation available for treatment in the private sector resulting in variance in medical cost and health care utilization.

Adjusted odds ratio indicated those with higher education to have higher propensity of multimorbidity in private facilities. One of the explanations could be, that, owing to higher health literacy level these patients consult health care providers more frequently thus increasing the probability of getting diagnosed with more conditions [18, 23]. The observed positive correlation between higher educational level and number of outpatient visits in our results substantiates this argument. We observed a strong association between increasing age and lower education in multimorbidity patients with increased healthcare visits in public facilities, whereas, among private multimorbidity patients, higher education was more associated with hospital visits. General hospitalization rates vary considerably with difference in income, education, and urban-rural residence [18, 23, 41].

Polypharmacy is one of the major documented patient care challenges in multimorbidity, since patients with multimorbidity consume significantly more medications, incur higher drug expenditures, are prone to more adverse effects and exhibit low treatment compliance [38, 39]. In our study we identified the factors responsible for increased number of medications consumed by multimorbidity patients for both facilities. In public facilities, among multimorbidity patients a fair association was seen between non-aboriginal status and medication use, whereas in private facilities better socio-economic condition was seen to be strongly associated with higher medication use. Chronic conditions require frequent medical consultations; patients with chronic illnesses often go to hospitals, which can increase the associated costs. A person with multimorbidity living in a rural area with limited income, however, continues to use traditional standard chronic public health services or not being able to seek specialist care; which causes inequality in healthcare use.

There is a paucity of publications on multimorbidity and equity worldwide [41]. Tackling this inequity remains essential if health inequalities are to be narrowed through the availability of effective health care. Similar findings such as sociodemographic and socioeconomic variables were found to be associated with multimorbidity in few other studies [42]. Rural areas had poorer quality of life with multimorbidities than their urban counterparts [43]. Generally, older age, female gender, low education and low-income people were seen to be likelier to have multimorbidity [44]. The effect of multimorbidity on females, the elderly, the low income and the vulnerable population is greater thus mandating that health services delivery should work towards achieving greater clinical care equity and universal health coverage for addressing multimorbidity.

The high prevalence of multimorbidity identified underlines the importance of current efforts to provide continuous, collaborative, patient-centered and comprehensive care at primary care. Policy decision-makers should pay attention to cost effective strategies based on early diagnosis and sensitization for a healthy diet, physical activity, no smoking and no alcohol. Developing primary care clinical practice guidelines on managing multimorbidity is an important component for the strengthening of the health system and to increase individual practitioner responsiveness to this challenge. Action is required to address the inequities for service provision for patients with multimorbidity at the public and private health systems. Further research is needed for the community, combining economic, social, cultural and ethnic characteristics, to enable a better understanding of the types of populations that are affected by multimorbidity. The knowledge gained from such research could inform strategies for the development of primary care models in India, which have to adapt to the challenge of meeting the needs of an increasingly older population with multimorbidity.

The major strength of the study is its large sample size and representativeness of primary care patient population in India. We elicited information on 21 chronic conditions which were selected through an iterative process. Our study covers all adult age groups and is nearly representative of users of both public and private health care facilities in terms of sex, age group, ethnicity and other socio-economic factors. The difference in distribution of the population structure is because of the nature of our study i.e. facility-based study.

The limitation of this study include first that, being a cross-sectional study no causal relationship can be established between socio-economic factors and multimorbidity. Since it is based on self-report, under- or over diagnosis of diseases and misclassification of disease status could not be excluded [45], especially among lower socio-economic group population. However, the amount of error may not have been substantial since studies have documented that estimates based on self-reports result in near to true prevalence. Similarly, simple disease counts predict morbidity burden equally as more complex measurement approaches to multimorbidity [46].

## Conclusion

Our study provides the first ever evidence on the emerging burden and inequities of multimorbidity in public and private primary health care settings in the Indian context. Targeted policies for health system planning should focus on workforce training, quality improvement strategies, development of clinical guidelines and quality indicators with regard to multimorbidity in primary care. Disadvantaged individuals with the same levels of multimorbidity require stronger financial protection. Investigating the occurrence of multimorbidity in deprived populations would lead to a better understanding of equity dimension of multimorbidity in future.

## Supplementary information

**Additional file 1.** Prevalence of chronic conditions and morbidity. Prevalence of single and multimodibity among public and private primary care.

**Additional file 2.** Comparison of the sample characteristics with actual population distribution in the state.

**Additional file 3.** Comparision of the sample population with the population of the state (census 2011).

## Data Availability

The datasets used and/or analysed during the current study are available from the corresponding author on reasonable request.

## Abbreviations

AOR: Adjusted odds ratio
APD: Acid peptic disease
APL: Above poverty line
BPL: Below poverty line
CHC: Community health center
CI: Confidence interval
IRR: Incidence rate ratio
LMIC: Low and medium income countries
NCD: Non communicable disease
PHQ: Patient health questionnaire
