# Supplementary material for: Magnitude and determinants of multimorbidity and health care utilization among patients attending public versus private primary care: a cross-sectional study from Odisha, India
Source: Int J Equity Health. 2020 Apr 29;19:57. doi: 10.1186/s12939-020-01170-y (PMC7191801; doi:10.1186/s12939-020-01170-y)
Supplement: Supplementary file 1 — Additional file 1. Prevalence of chronic conditions and morbidity. Prevalence of single and multimodibity among public and private primary care. [file 12939_2020_1170_MOESM1_ESM.docx]

**Additional file 1- Sampling strategies**

Random selection

Random selection

From each facility nearly 70 patients were selected using systematic random sampling from the outpatient register

KBK+

Districts (11)

90

Non KBK

Districts (19)

4 Districts*

6 Districts*

2 public and 2 private facilities from each district^#^

2 public and 2 private facilities from each district

Total 20 public and 20 private facilities were selected

Odisha State (Total 30 districts)

*Each Districts have 6-8 CHCs (public facilities)

#for each public facilities one private facility form the same locality was selected
