# Supplementary material for: Magnitude and determinants of multimorbidity and health care utilization among patients attending public versus private primary care: a cross-sectional study from Odisha, India
Source: Int J Equity Health. 2020 Apr 29;19:57. doi: 10.1186/s12939-020-01170-y (PMC7191801; doi:10.1186/s12939-020-01170-y)
Supplement: Supplementary file 2 — Additional file 2. Comparison of the sample characteristics with actual population distribution in the state. [file 12939_2020_1170_MOESM2_ESM.docx]

**Additional file 2- Prevalence of chronic conditions and morbidity**

| Chronic condition | Total (n=1649) | Public (n=849) | | Private (n=840) | Chi-sqaure  p value |
| --- | --- | --- | --- | --- | --- |
|  | Weighted %  [95% CI] | Weighted %  [95% CI] | | Weighted %  [95% CI] |  |
| Acid Peptic Disorder | 30.8[28.5-33.2] | 32.3[29.1-35.6] | | 28.3[24.8-31.8] | 0.047* |
| Hypertension | 16.4[14.6-18.4] | 14.8[12.3-17.3] | | 18.9[15.8-22.0] | 0.258 |
| Arthritis | 15.4[13.6-17.4] | 16.9[14.3-19.5] | | 13.0[10.4-15.6] | 0.007* |
| Chronic Back pain | 11.7 [10.1-13.5] | 13.5[11.1-15.9] | | 8.8[6.7-9.8] | 0.004* |
| Vision problem/blindness | 6.5[5.3-7.9] | 7.1[5.3-8.8] | | 5.6[3.9-7.3] | 0.276 |
| Diabetes | 6.4[5.3-7.8] | 5.5[4.0-7.1] | | 7.8[5.7-9.9] | 0.185 |
| Chronic Lung Disease | 2.8[2.1-3.8] | 2.2[1.2-3.2] | | 3.7[2.2-5.2] | 0.124 |
| Tuberculosis | 1.5[0.9-2.3] | 2.0[1.0-2.9] | | 0.6[0.1-1.2] | 0.045* |
| Deafness | 1.3[0.8-2.0] | 1.6[0.7-2.4] | | 0.8[0.2-1.4] | 0.161 |
| Hypotension | 0.9[0.5-1.4] | 1[0.9-11.0] | | 0.7[6.3-7.7] | 0.543 |
| Thyroid Disease | 0.8[0.4-1.4] | 0.9[0.16-1.6] | | 0.62[0.01-1.2] | 0.839 |
| Depression | 0.7[0.4-1.3] | 1.2[0.78-2.1] | | 0[0] | 0.0028 |
| Heart Disease | 0.7[0.4-1.3] | 0.2[-0.03-0.5] | | 0.1[0.4-2.4] | 0.065 |
| Filariasis | 0.6[0.3-1.1] | 0.5[0.01-1.0] | | 0.63[0.01-1.2] | 0.925 |
| Eczema | 0.6[0.3-1.0] | 0.8 [0.5-1.1] | | 0.2 [0.05-0.4] | 0.043* |
| Dementia | 0.4[0.2-0.9] | 0.5[0.01-0.90] | | 0.30[-0.01 to 0.7] | 0.764 |
| Psoriasis | 0.3[0.1-0.7] | 0[0] | | 0.8[0.53-1.1] | 0.011* |
| Kidney disease | 0.3[0.1-0.7] | 0.01[-0.01 to 0.2] | | 0.6[-0.01 to 1.2] | 0.158 |
| Alcohol disorder | 0.3[0.1-0.7] | 0.24[-0.01to 0.6] | | 0.4[-0.01 to 0.8] | 0.607 |
| Stroke | 0.3[0.1-0.8] | 0.22[-0.11 to 0.5] | | 0.4[-0.18 to 0.9] | 0.953 |
| **Morbidities** |  |  |  |  |  |
| Zero morbidity | 45.3[42.7-47.8] | 44.2[40.8-47.7] | | 46.6[43.1-50.6] | 0.004* |
| One morbidity | 26.4[24.1-28.7] | 25.1[22.0-28.1] | | 28.5[25.1-31.9] |  |
| Two morbidities | 16.3[14.4-18.3] | 18.1[15.4-20.8] | | 13.6[10.9-16.3] |  |
| >Three Morbidities | 12.0[10.3-13.7] | 12.6[10.3-14.9] | | 11.0[8.5-13.6] |  |

*p value < 0.05
