# Supplementary material for: Magnitude and determinants of multimorbidity and health care utilization among patients attending public versus private primary care: a cross-sectional study from Odisha, India
Source: Int J Equity Health. 2020 Apr 29;19:57. doi: 10.1186/s12939-020-01170-y (PMC7191801; doi:10.1186/s12939-020-01170-y)
Supplement: Supplementary file 3 — Additional file 3. Comparision of the sample population with the population of the state (census 2011). [file 12939_2020_1170_MOESM3_ESM.docx]

**Additional file 3- Comparision of the sample population with the population of the state (census 2011)**

|  | State (%) | Sample (%) |
| --- | --- | --- |
| % of female | 49.46 | 44.2 |
| **Age group** |  |  |
| 18-29 | 20.7 | 22.6 |
| 30-39 | 14.7 | 18.1 |
| 40-49 | 12.0 | 20.5 |
| 50-59 | 9.0 | 16.7 |
| 60-69 | 5.8 | 14.6 |
| >=70 | 3.4 | 7.5 |
| **Socio-economic status^#^** |  |  |
| BPL | 33.7 | 38.4 |
| **Ethnicity** |  |  |
| SC/ST | 40 | 28.0[25.7-30.3] |
| **Literacy status** |  |  |
| Literate | 73 | 63 |

#Socio-economic survey, 2012-13, Odisha.

Reference: Census 2011, India

Available at : <http://www.censusindia.gov.in/2011-prov-results/prov_data_products_odisha.html>
